# Radiotherapy to the primary tumour for newly diagnosed, metastatic prostate cancer (STAMPEDE): a randomised controlled phase 3 trial

**DOI:** 10.1016/S0140-6736(18)32486-3

**Published:** 2018-12-01

**Authors:** Christopher C Parker, Nicholas D James, Christopher D Brawley, Noel W Clarke, Alex P Hoyle, Adnan Ali, Alastair W S Ritchie, Gerhardt Attard, Simon Chowdhury, William Cross, David P Dearnaley, Silke Gillessen, Clare Gilson, Robert J Jones, Ruth E Langley, Zafar I Malik, Malcolm D Mason, David Matheson, Robin Millman, J Martin Russell, George N Thalmann, Claire L Amos, Roberto Alonzi, Amit Bahl, Alison Birtle, Omar Din, Hassan Douis, Chinnamani Eswar, Joanna Gale, Melissa R Gannon, Sai Jonnada, Sara Khaksar, Jason F Lester, Joe M O'Sullivan, Omi A Parikh, Ian D Pedley, Delia M Pudney, Denise J Sheehan, Narayanan Nair Srihari, Anna T H Tran, Mahesh K B Parmar, Matthew R Sydes, Systemic Therapy for Advanced or Metastatic Prostate cancer: Evaluation of Drug Efficacy (STAMPEDE) investigators

**Affiliations:** a Academic Urology Unit, Royal Marsden Hospital, London, UK; b Institute of Cancer Research, London, UK; c Institute of Cancer and Genomic Sciences, University of Birmingham, Birmingham, UK; d Medical Research Council (MRC) Clinical Trials Unit at University College London (UCL), London, UK; e Genito-Urinary Cancer Research Group, Department of Surgery, The Christie Hospital, Manchester, UK; f Department of Urology, Salford Royal Hospitals, Manchester, UK; g Department of Clinical Oncology, The Christie NHS Foundation Trust, Manchester, UK; h The FASTMAN/Genito-Urinary Cancer Research Groups, Division of Cancer Sciences, and Belfast–Manchester Movember Centre of Excellence, Manchester Cancer Research Centre, University of Manchester, Manchester, UK; i UCL Cancer Institute, UCL, London, UK; j Department of Medical Oncology, Guy's & St Thomas' NHS Foundation Trust, London, UK; k Department of Urology, St James University Hospital, Leeds, UK; l Division of Cancer Sciences, University of Manchester and the Christie, Manchester, UK; m Division of Oncology and Haematology, Kantonsspital, St Gallen, Switzerland; n Beatson West of Scotland Cancer Centre, University of Glasgow, Glasgow, UK; o Institute of Cancer Sciences, University of Glasgow, Glasgow, UK; p The Clatterbridge Cancer Centre NHS Foundation Trust, Liverpool, UK; q Division of Cancer & Genetics, School of Medicine, Cardiff University, Cardiff, UK; r Faculty of Education, Health and Wellbeing, University of Wolverhampton, Wolverhampton, UK; s Department of Urology, University Hospital, Inselspital, Bern, Switzerland; t Mount Vernon Cancer Centre, London, UK; u Bristol Haematology and Oncology Centre, Bristol, UK; v Rosemere Cancer Centre, Lancashire Teaching Hospitals, Preston, UK; w School of Cancer Sciences, University of Manchester, Manchester, UK; x Department of Clinical Oncology, Weston Park Cancer Centre, Sheffield Teaching Hospitals NHS Foundation Trust, Sheffield, UK; y Department of Radiology, University Hospital Birmingham NHS Foundation Trust, Birmingham, UK; z Portsmouth Oncology Centre, Queen Alexandra Hospital, Portsmouth, UK; aa Department of Health Services Research, London School of Hygiene & Tropical Medicine, London, UK; ab Department of Oncology, Gloucestershire Hospitals NHS Foundation Trust, Gloucester, UK; ac St Luke's Cancer Centre, Royal Surrey County Hospital, Guildford, UK; ad Velindre Cancer Centre, Cardiff, UK; ae Centre for Cancer Research and Cell Biology, Queen's University Belfast, Belfast, UK; af Department of Clinical Oncology, East Lancashire Hospitals NHS Trust, Blackburn, UK; ag Northern Centre for Cancer Care, Freeman Hospital, Newcastle upon Tyne, UK; ah Clinical Oncology, Singleton Hospital, Swansea, UK; ai Exeter Oncology Centre, Royal Devon & Exeter Hospital, Exeter, UK; aj Department of Oncology, Shrewsbury and Telford Hospitals NHS Trust, Shrewsbury, UK

## Abstract

**Background:**

Based on previous findings, we hypothesised that radiotherapy to the prostate would improve overall survival in men with metastatic prostate cancer, and that the benefit would be greatest in patients with a low metastatic burden. We aimed to compare standard of care for metastatic prostate cancer, with and without radiotherapy.

**Methods:**

We did a randomised controlled phase 3 trial at 117 hospitals in Switzerland and the UK. Eligible patients had newly diagnosed metastatic prostate cancer. We randomly allocated patients open-label in a 1:1 ratio to standard of care (control group) or standard of care and radiotherapy (radiotherapy group). Randomisation was stratified by hospital, age at randomisation, nodal involvement, WHO performance status, planned androgen deprivation therapy, planned docetaxel use (from December, 2015), and regular aspirin or non-steroidal anti-inflammatory drug use. Standard of care was lifelong androgen deprivation therapy, with up-front docetaxel permitted from December, 2015. Men allocated radiotherapy received either a daily (55 Gy in 20 fractions over 4 weeks) or weekly (36 Gy in six fractions over 6 weeks) schedule that was nominated before randomisation. The primary outcome was overall survival, measured as the number of deaths; this analysis had 90% power with a one-sided α of 2·5% for a hazard ratio (HR) of 0·75. Secondary outcomes were failure-free survival, progression-free survival, metastatic progression-free survival, prostate cancer-specific survival, and symptomatic local event-free survival. Analyses used Cox proportional hazards and flexible parametric models, adjusted for stratification factors. The primary outcome analysis was by intention to treat. Two prespecified subgroup analyses tested the effects of prostate radiotherapy by baseline metastatic burden and radiotherapy schedule. This trial is registered with ClinicalTrials.gov, number NCT00268476.

**Findings:**

Between Jan 22, 2013, and Sept 2, 2016, 2061 men underwent randomisation, 1029 were allocated the control and 1032 radiotherapy. Allocated groups were balanced, with a median age of 68 years (IQR 63–73) and median amount of prostate-specific antigen of 97 ng/mL (33–315). 367 (18%) patients received early docetaxel. 1082 (52%) participants nominated the daily radiotherapy schedule before randomisation and 979 (48%) the weekly schedule. 819 (40%) men had a low metastatic burden, 1120 (54%) had a high metastatic burden, and the metastatic burden was unknown for 122 (6%). Radiotherapy improved failure-free survival (HR 0·76, 95% CI 0·68–0·84; p<0·0001) but not overall survival (0·92, 0·80–1·06; p=0·266). Radiotherapy was well tolerated, with 48 (5%) adverse events (Radiation Therapy Oncology Group grade 3–4) reported during radiotherapy and 37 (4%) after radiotherapy. The proportion reporting at least one severe adverse event (Common Terminology Criteria for Adverse Events grade 3 or worse) was similar by treatment group in the safety population (398 [38%] with control and 380 [39%] with radiotherapy).

**Interpretation:**

Radiotherapy to the prostate did not improve overall survival for unselected patients with newly diagnosed metastatic prostate cancer.

**Funding:**

Cancer Research UK, UK Medical Research Council, Swiss Group for Clinical Cancer Research, Astellas, Clovis Oncology, Janssen, Novartis, Pfizer, and Sanofi-Aventis.

## Introduction

Patients with metastatic cancer typically receive systemic treatment, with local therapy reserved—if required—for symptom palliation. However, local treatment to the primary tumour might be more useful than previously appreciated. In animal models of cancer, primary tumours metastasise not merely by disseminating tumour cells into the circulation but also by priming the premetastatic niche.^1^ Proliferation of tumour cells at distant sites to form overt metastases is dependent on compounds secreted by the primary tumour into the circulation.^2^ In these models, local treatment of the primary tumour inhibits not just the initiation of distant disease but also the progression of existing metastases.

**Research in context**

**Evidence before this study**

We searched MEDLINE (1966–2018), Embase (1982–2018), trial registers (Cochrane Central Register of Controlled Trials and ClinicalTrials.gov), and major urology and oncology conference proceedings (1990–2018) to retrieve randomised controlled trials of radiotherapy in metastatic prostate cancer. The search strategy included a range of terms to identify randomised controlled trials, prostate cancer, and radiotherapy. One relevant trial—HORRAD—was identified (n=432, 270 deaths) in which no evidence was reported of an overall survival benefit for prostate radiotherapy (hazard ratio [HR] 0·90, 95% CI 0·70–1·14), but a hypothesis was generated that survival might be improved in a subgroup of patients with low metastatic burden (HR 0·68, 95% CI 0·42–1·10).

**Added value of this study**

To the best of our knowledge, our large randomised trial (n=2061, 761 deaths) provides the best available evidence about the role of prostate radiotherapy in metastatic prostate cancer. Our findings showed no overall survival benefit of radiotherapy to the prostate in men with newly diagnosed prostate cancer. However, a subgroup analysis supported the hypothesis of HORRAD, that prostate radiotherapy improves survival in men with low metastatic burden.

**Implications of all the available evidence**

Evidence suggests that prostate radiotherapy improves overall survival for men with metastatic prostate cancer who have a low metastatic burden, but not for unselected patients. Prostate radiotherapy should be a standard treatment option for men with newly diagnosed disease with a low metastatic burden.

Radical local treatment of the primary tumour has been tested in several randomised controlled trials in patients with metastatic cancer. Cytoreductive nephrectomy improved survival in patients with metastatic renal carcinoma,^3^, ^4^ but this benefit was not confirmed in a more recent trial in patients with advanced disease.^5^ Radiotherapy to the primary tumour has not been shown to improve survival in patients with metastatic small-cell lung cancer^6^ or metastatic breast cancer,^7^ but these trials were relatively small and might not have detected a modest, but worthwhile, benefit.

In men with metastatic prostate cancer, retrospective analyses have noted an association between use of radiotherapy to the primary tumour and improved overall survival.^8^, ^9^, ^10^, ^11^ The survival benefit associated with prostate radiotherapy was reported to be greater in patients with a better prognosis.^8^, ^9^, ^11^ The HORRAD trial randomised 432 men with metastatic prostate cancer to androgen deprivation therapy with or without prostate radiotherapy and found no evidence of an overall survival benefit (hazard ratio [HR] 0·90, 95% CI 0·70–1·14) but raised the possibility that survival might be improved in a subgroup of patients with fewer than five bone metastases (0·68, 0·42–1·10).^12^

We hypothesised that radical radiotherapy to the prostate would improve overall survival in men presenting with metastatic prostate cancer and that the survival benefit would be greatest in men with a low metastatic burden.

## Methods

### Study design and participants

We did a randomised controlled phase 3 trial at 117 hospitals in Switzerland and the UK. Eligible patients had prostate cancer that was newly diagnosed, with no previous radical treatment, and had metastatic disease confirmed on a bone scintigraphic scan and soft-tissue imaging done within 12 weeks of starting androgen deprivation therapy. All patients were intended for long-term androgen deprivation therapy and started treatment no earlier than 12 weeks before randomisation. There were no age restrictions; patients were required to have no contraindications to radiotherapy and no clinically significant cardiovascular history.

This trial was done in accordance with Good Clinical Practice guidelines and the Declaration of Helsinki and had relevant regulatory and ethics approvals. All patients gave written informed consent. The rationale and design have been described previously.^13^ Full details are in the protocol.

### Randomisation and masking

Patients were randomised centrally using a computerised algorithm, which was developed and maintained by the Medical Research Council (MRC) Clinical Trials Unit at University College London. Minimisation with a random element of 20% was used, stratifying for hospital, age at randomisation (<70 years *vs* ≥70 years), nodal involvement (negative *vs* positive *vs* indeterminate), WHO performance status (0 *vs* 1 or 2), planned androgen deprivation therapy, and regular aspirin or non-steroidal anti-inflammatory drug use (yes or no). Planned docetaxel use was added as a stratification factor on Dec 17, 2015. Allocation was 1:1 to either standard of care (control) or standard of care and radiotherapy (radiotherapy). Patients and clinical and study staff were aware of the treatment allocation for practical reasons, and the key efficacy outcome measures were objective.

### Procedures

All patients received lifelong androgen deprivation therapy as either gonadotrophin-releasing hormone agonists or antagonists or orchidectomy. Docetaxel was permitted in addition to hormone therapy after its approval in the UK on Dec 17, 2015. Docetaxel, when used, was given as six 3-weekly cycles of 75 mg/m^2^, with or without prednisolone 10 mg daily.

External-beam radiotherapy to the prostate was given as one of two schedules nominated before randomisation: either 36 Gy in six consecutive weekly fractions of 6 Gy, or 55 Gy in 20 daily fractions of 2·75 Gy over 4 weeks. Radiotherapy was given with the patient supine and with a full bladder and an empty rectum. The planning target volume consisted of the prostate only, with an 8 mm margin posteriorly and a 10 mm margin elsewhere. Radiotherapy was to commence as soon as practicable after randomisation, and within 3–4 weeks after the last docetaxel dose.

Patients were followed up every 6 weeks until 6 months after randomisation, then every 12 weeks to 2 years, then every 6 months to 5 years, then annually thereafter. Prostate-specific antigen (PSA) levels were measured at every follow-up visit; further tests were at the clinician's discretion. Nadir PSA was the lowest level of PSA reported within 24 weeks after randomisation. Toxic effects and symptoms were reported at regular follow-up visits or when an adverse event was categorised as serious. Adverse events were graded with the National Cancer Institute Common Terminology Criteria for Adverse Events (CTCAE) version 4.0. Adverse effects on the bowel and bladder during radiotherapy, and possible long-term effects of radiotherapy, were recorded separately in patients assigned standard of care and radiotherapy using the Radiation Therapy Oncology Group (RTOG) scale.^14^

Metastatic burden at randomisation was assessed through whole-body scintigraphy and CT or MRI staging scans. Scans were centralised and reviewed by one of us (AA), with 10% independent review by a radiologist (HD). The metastatic burden was classified according to the definition used in the CHAARTED trial:^15^ high metastatic burden was defined as four or more bone metastases with one or more outside the vertebral bodies or pelvis, or visceral metastases, or both; all other assessable patients were considered to have low metastatic burden.

### Outcomes

The primary efficacy outcome was overall survival, defined as time from randomisation to death from any cause. Failure-free survival was the primary activity outcome measure for interim analyses and was defined as time from randomisation to first evidence of at least one of: biochemical failure; progression either locally, in lymph nodes, or in distant metastases; or death from prostate cancer. Biochemical failure was based on a rise above the lowest PSA value reported within 24 weeks after enrolment of 50% and to at least 4 ng/mL; patients without a fall of 50% were considered to have biochemical failure at time zero. Secondary outcomes were progression-free survival (defined as failure-free survival but without biochemical events) and metastatic progression-free survival (defined as time from randomisation to new metastases or progression of existing metastases or death). Cause of death was determined by the site investigator, with some causes reclassified as prostate cancer according to predefined criteria that indicated prostate cancer to be the likely cause. Symptomatic local events were defined as any of the following: urinary-tract infection, new urinary catheterisation, acute kidney injury, transurethral resection of the prostate, urinary-tract obstruction, ureteric stent, nephrostomy, colostomy, and surgery for bowel obstruction. Patients without the event of interest were censored at the time last known to be event-free.

### Statistical analysis

This randomised comparison was incorporated within the Systemic Therapy for Advanced or Metastatic Prostate cancer: Evaluation of Drug Efficacy (STAMPEDE) multiarm multistage (MAMS) platform protocol (appendix p 6). It was designed with a seamless phase 2/3 approach.^16^ The sample size was calculated using *nstage* and its predecessor programs in Stata, which enable design of MAMS trials.^17^ Assuming, for the control group, a median failure-free survival of roughly 1 year and median survival of about 3·5 years, we targeted a 25% relative improvement (HR 0·75) in both failure-free survival and overall survival for the group allocated radiotherapy to the prostate over the control group.

For the efficacy stage analysis of the pairwise comparison of standard of care and radiotherapy versus standard of care for overall survival, approximately 267 deaths in patients allocated to the control group were needed for 90% power and a one-sided α of 2·5%, accounting for three intermediate analyses of failure-free survival (analysed June, 2014, November, 2014, and May, 2015). For this comparison, the pairwise and family-wise error rates were judged very similar, because of the limited overlap in events with other reported comparisons from the protocol and the non-binding nature of the interim analyses.

The initial sample size target was 1250 patients. During the trial, weekly and daily radiotherapy schedules were nominated approximately equally. Therefore, the sample size was increased to roughly 1800, without reference to outcome data, to provide good power for failure-free survival in each radiotherapy schedule-defined subgroup when the comparison reached its target power overall, assuming that the effect of radiotherapy would be the same regardless of schedule. We predicted about 300 failure-free survival events in the control group on each schedule at the time of the main analysis, which would provide approximately 90% power with a one-sided α of 0·015 to detect an HR of 0·75. The effect of radiotherapy on survival within a nominated radiotherapy schedule would be investigated if there was both an effect on failure-free survival and 200 or more deaths in the control group were reported for that nominated schedule.

In May, 2018, based on accumulating external data and without reference to any data from this comparison in STAMPEDE, we prespecified that any effect from radiotherapy would be greatest in patients with a low baseline metastatic burden and that this hypothesis could be tested with reasonable power, regardless of interaction test results. If roughly 40% of patients had a low metastatic burden, we anticipated more than 90% power for failure-free survival (HR 0·70) if median failure-free survival were 24 months in the control group and about 60% power for overall survival (HR 0·70) if median survival were roughly 6 years. If about 60% of patients had a high metastatic burden, we anticipated the subgroup analysis would have roughly 88% power for failure-free survival (HR 0·80) if median failure-free survival were 12 months in the control group and about 63% power for overall survival (HR 0·80) if median survival were 4 years.

Standard survival analysis methods were used to analyse time-to-event data in Stata version 15. A non-parametric stratified log-rank test was used to detect a difference in survival between treatment groups; this analysis was stratified across the minimisation factors used at randomisation (except hospital and planned androgen deprivation therapy) plus protocol-specific periods defined by other arms recruiting to STAMPEDE or changes to standard of care that could affect the population being randomised. Cox proportional hazards regression models adjusting for the same stratification factors and stratified by time were used to estimate relative treatment effects. An HR less than 1·00 favoured radiotherapy. Flexible parametric models were fitted with degrees of freedom (5,5) and adjusted for stratification factors and time.^18^ Medians and 3-year survival estimates are presented from the flexible parametric models fitted to the data; these are more reliable than reading the Kaplan-Meier curves; graphs show estimated survival over time from both. The proportional hazards assumption was tested; restricted mean survival time was emphasised in the presence of non-proportionality, using a t-star of 59 months as determined by the Royston and Parmar method.^18^ Cause-specific and Fine and Gray regression models^19^ were used for competing risk analysis of prostate cancer-specific, lymph node, and metastatic progression-free and symptomatic local event-free survival. All tests are presented as two-sided, with 95% CIs and relevant p values.

Subgroup analyses were prespecified for the nominated radiotherapy schedule (daily *vs* weekly) and for baseline metastatic burden (low *vs* high), when determinable. Exploratory interaction analyses considered the consistency of treatment effect within stratification factors, by time, by Gleason score, and by PSA before hormone therapy.

Median follow-up was ascertained by reverse-censoring on death. All patients were included in the primary efficacy analysis according to allocated treatment, and the analysis was done on an intention-to-treat basis. Adverse event data are shown for the safety population, which consisted of patients with at least one follow-up assessment analysed according to the treatment approach started; patients were excluded if they had no adverse event data. A sensitivity analysis was done on an intention-to-treat basis. Data for symptomatic local events are also presented. All other analyses are exploratory.

Accumulating interim data were reviewed by an Independent Data Monitoring Committee, guided by lack-of-benefit stopping guidelines.

This trial is registered at ClinicalTrials.gov (number NCT00268476) and ISRCTN.com (ISRCTN78818544).

### Role of the funding source

MRC employees contributed to study design, data collection, data analysis, data interpretation, and writing of this report. CDB, MRS, APH, and AA had access to raw data. The corresponding author had final responsibility for the decision to submit for publication.

## Results

**Figure 1:**
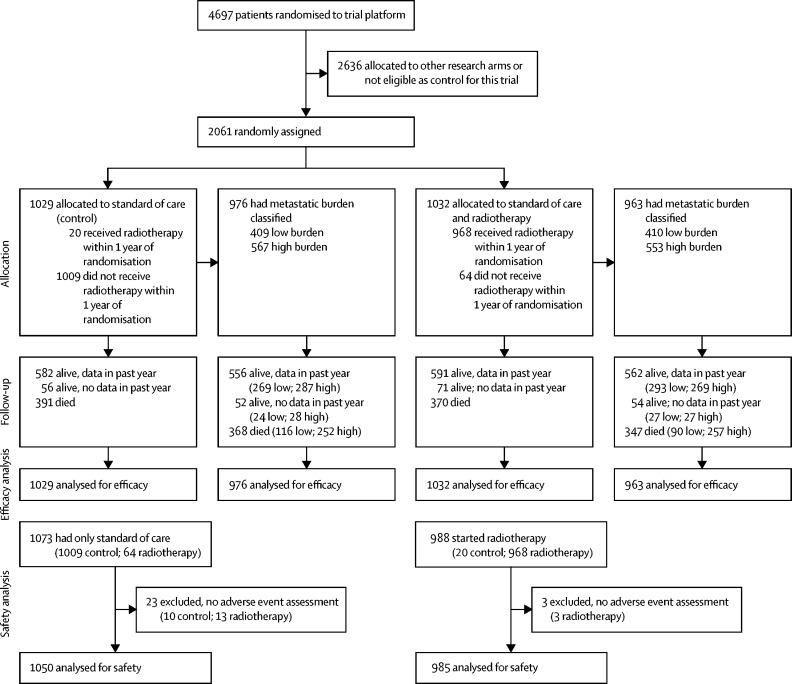
Trial profile

Between Jan 22, 2013, and Sept 2, 2016, 2061 patients were randomly allocated either standard of care (control group, n=1029) or standard of care and radiotherapy (radiotherapy group, n=1032; figure 1). Groups were well balanced with respect to baseline characteristics (table 1). Median age was 68 years (IQR 63–73) and median PSA before androgen deprivation therapy was 97 ng/mL (33–315). 1630 (79%) patients had a Gleason score of 8–10. 1836 (89%) had bone metastases. 1466 (71%) had a WHO performance status of zero. Baseline characteristics of 1939 (94%) patients in whom metastatic disease burden could be determined are shown in the appendix (p 1).

**Table 1:** Baseline characteristics

		**Control (n=1029)**	**Radiotherapy (n=1032)**
Age at randomisation (years)		68 (63–73)	68 (63–73)
	Range	37–86	45–87
WHO performance status			
	0	732 (71%)	734 (71%)
	1–2	297 (29%)	298 (29%)
Pain from prostate cancer			
	Absent	820 (81%)	844 (83%)
	Present	198 (19%)	170 (17%)
	Missing data	11	18
Previous notable health issues			
	Myocardial infarction	67 (7%)	57 (6%)
	Cerebrovascular disease	29 (3%)	30 (3%)
	Congestive heart failure	5 (<1%)	8 (1%)
	Angina	46 (4%)	51 (5%)
	Hypertension	408 (40%)	440 (43%)
	Missing data	5	8
T category at randomisation			
	T0	0 (0%)	2 (<1%)
	T1	12 (1%)	12 (1%)
	T2	84 (9%)	89 (9%)
	T3	585 (62%)	603 (63%)
	T4	260 (28%)	246 (26%)
	TX	88	80
N category at randomisation			
	N0	345 (36%)	344 (36%)
	N+	620 (64%)	620 (64%)
	NX	64	68
Metastatic burden			
	Low	409 (42%)	410 (43%)
	High	567 (58%)	553 (57%)
	Not classified	53	69
Sites of metastases			
	Bone	919 (89%)	917 (89%)
	Liver	23 (2%)	19 (2%)
	Lung	42 (4%)	48 (5%)
	Distant lymph nodes	294 (29%)	304 (29%)
	Other	35 (3%)	33 (3%)
Gleason sum score			
	≤7	173 (17%)	172 (18%)
	8–10	820 (83%)	810 (82%)
	Unknown	36	50
PSA before androgen deprivation therapy (ng/mL)		98 (30–316)	97 (33–313)
	Range	1–20 590	1–11 156
Time from diagnosis (days)		73 (55–94)	73 (55–93)
	Missing data	7	16
Time from starting hormones (days)		52 (35–70)	55 (34–70)
	Range	−32 to 84	−10 to 85
	Missing data	0	1
Planned docetaxel			
	No	845 (82%)	849 (82%)
	Yes	184 (18%)	183 (18%)
Nominated radiotherapy schedule*			
	Weekly	482 (47%)	497 (48%)
	Daily	547 (53%)	535 (52%)

Data are median (IQR) or n (%), unless otherwise stated. PSA=prostate-specific antigen.

* The weekly schedule was 36 Gy in six fractions over 6 weeks and the daily schedule was 55 Gy in 20 fractions over 4 weeks.

Standard hormone therapy was luteinising hormone-releasing hormone analogues for 2046 (99%) men. Standard of care included docetaxel for 367 (18%) patients. Of 2061 patients undergoing random assignment, roughly half were nominated for each radiotherapy schedule, with 979 (48%) nominating the weekly schedule and 1082 (52%) the daily schedule. Of 968 patients assigned radiotherapy who started radiotherapy within 1 year after randomisation, 906 received their planned schedule and 62 received the alternative or another schedule. Two patients assigned radiotherapy received their planned schedule later than 1 year after randomisation, and 62 did not receive radiotherapy at all (mainly because of patient's choice). In patients who started radiotherapy, median time to starting radiotherapy was 35 days (IQR 28–60) after randomisation, and 95 days (74–120) from starting hormone therapy (most patients started androgen deprivation therapy before randomisation; appendix p 2). Only 20 (2%) patients allocated to the control group received radiotherapy within 1 year of randomisation.

**Figure 2:**
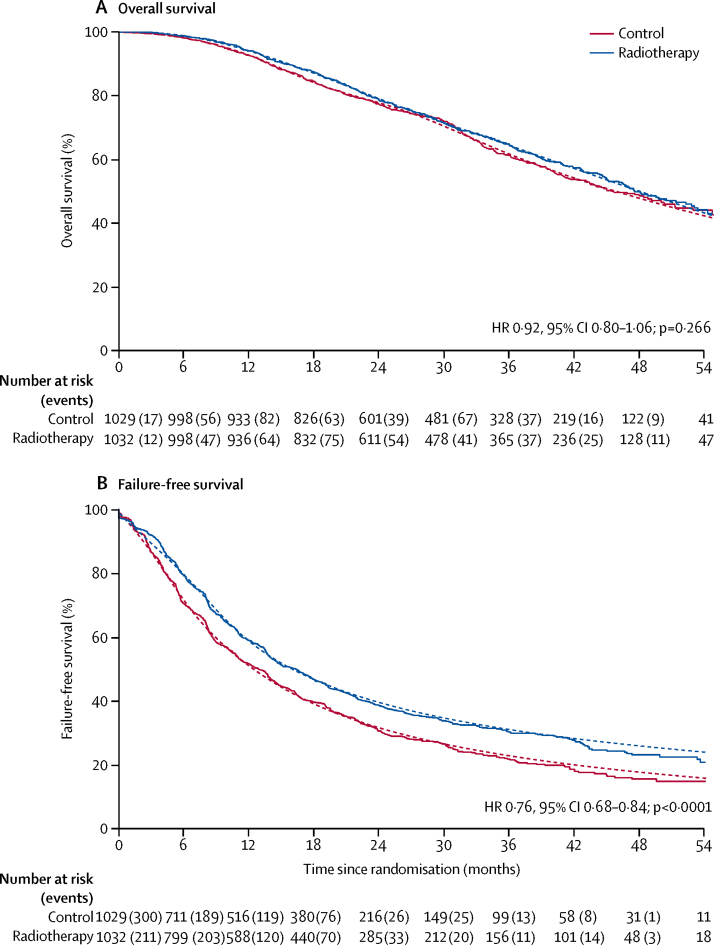
Overall survival and failure-free survival, by treatment HR=hazard ratio. Solid lines show the Kaplan-Meier analysis and dotted lines show the flexible parametric model.

Median follow-up was 37 months (IQR 24–48). 391 patients assigned to the control group died (median survival 46 months [IQR 27–not reached]; 3-year survival 62%). Compared with controls, no survival advantage was noted with radiotherapy (stratified log-rank test p=0·451; HR 0·92, 95% CI 0·80–1·06; p=0·266), with 370 deaths in the radiotherapy group (median survival 48 months [IQR 27–not reached]; 3-year survival 65%; figure 2A; table 2). There was no evidence of non-proportional hazards.

**Table 2:** Summary of estimated treatment effect for main outcome measures, for all patients and by metastatic burden

	**Adjusted hazard ratio (95% CI)**	Survival at 3 years*		Restricted mean survival time (months)*		
		Control	Radiotherapy	Control	Radiotherapy	Difference (95% CI)
**Overall survival**						
All patients	0·92 (0·80–1·06)	62%	65%	41·6	42·5	1·0 (−0·6 to 2·5)
Low metastatic burden	0·68 (0·52–0·90)	73%	81%	45·4	49·1	3·6 (1·0 to 6·2)
High metastatic burden	1·07 (0·90–1·28)	54%	53%	38·8	37·6	−1·2 (−3·5 to 1·1)
**Failure-free survival**						
All patients	0·76 (0·68–0·84)	23%	32%	21·4	26·2	4·8 (2·8 to 6·7)
Low metastatic burden	0·59 (0·49–0·72)	33%	50%	27·4	36·1	8·6 (5·6 to 11·7)
High metastatic burden	0·88 (0·77–1·01)	17%	18%	17·3	18·8	1·5 (−0·7 to 3·6)
**Progression-free survival**						
All patients	0·96 (0·85–1·08)	44%	44%	32·4	33·1	0·7 (−0·9 to 2·3)
Low metastatic burden	0·78 (0·63–0·98)	58%	63%	39·4	42·9	3·5 (0·4 to 6·7)
High metastatic burden	1·09 (0·94–1·26)	35%	30%	28·0	26·2	−1·8 (−4·3 to 0·8)
**Metastatic progression-free survival**						
All patients	0·97 (0·86–1·10)	47%	47%	33·9	34·4	0·4 (−1·5 to 2·4)
Low metastatic burden	0·80 (0·63–1·01)	62%	67%	41·1	44·2	3·1 (0·2 to 6·0)
High metastatic burden	1·10 (0·95–1·28)	37%	33%	29·3	27·3	−2·0 (−4·7 to 0·7)
**Prostate cancer-specific survival**						
All patients†	0·93 (0·80–1·09)	66%	69%	43·9	44·6	0·7 (−1·1 to 2·5)
Low metastatic burden	0·65 (0·47–0·90)	79%	86%	48·6	51·8	3·3 (1·0 to 5·5)
High metastatic burden	1·10 (0·92–1·32)	58%	56%	40·6	39·0	−1·6 (−3·9 to 0·7)
**Symptomatic local event-free survival**						
All patients	1·07 (0·93–1·22)	57%	55%	38·2	37·2	−1·1 (−3·1 to 0·9)
Low metastatic burden	0·82 (0·64–1·05)	65%	72%	41·6	44·0	2·4 (−0·7 to 5·4)
High metastatic burden	1·23 (1·05–1·46)	50%	43%	35·8	32·2	−3·6 (−6·2 to −1·0)

Hazard ratio and restricted mean survival time differences are for radiotherapy relative to control.

* Survival probabilities and restricted mean survival time estimates are taken from flexible parametric models (t-star, 59 months).

† Competing risks analysis, sub-hazard ratio 0·94, 95% CI 0·81–1·10; p=0·431.

**Figure 3:**
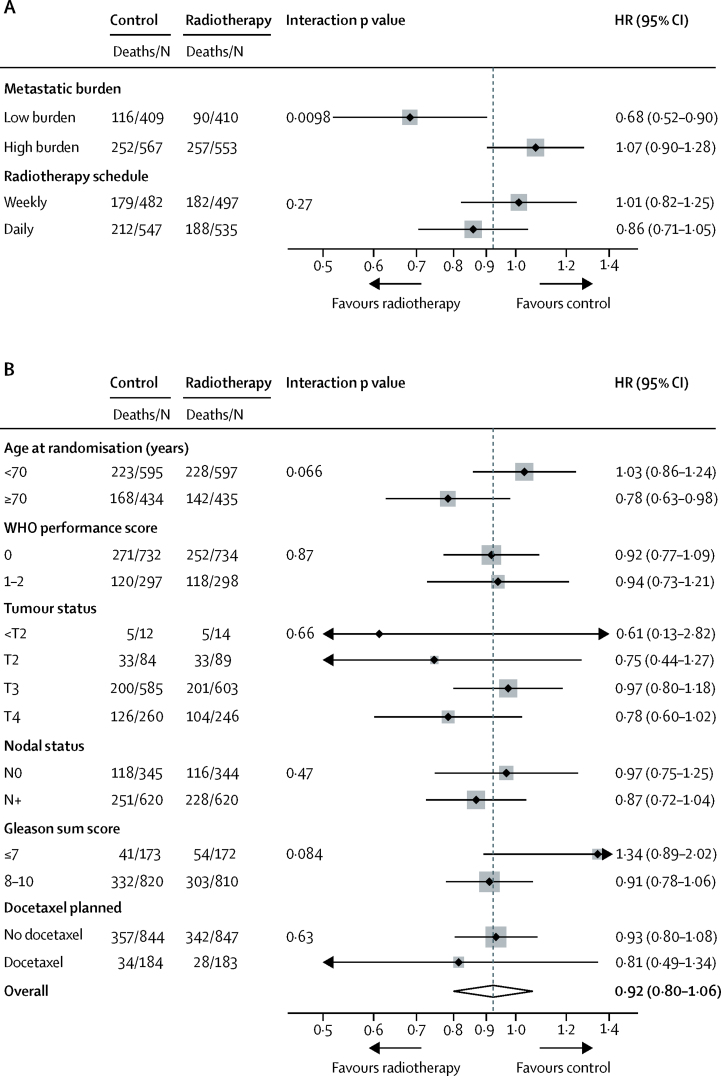
Treatment effect on overall survival within selected baseline categories HR=hazard ratio. PSA before androgen deprivation therapy (continuous), p=0·029; effect of adding radiotherapy is smaller with higher PSA. Patients with unknown T stage (TX), unknown N category (NX), or unknown Gleason sum score are not presented in the forest plot and do not contribute to interaction test results. Dotted line shows the overall hazard ratio. (A) Prespecified subgroup analyses. (B) Exploratory subgroup analyses.

**Figure 4:**
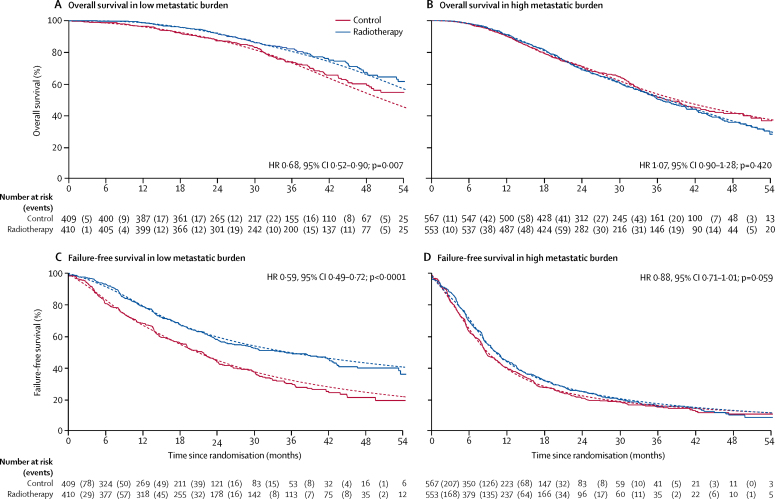
Overall survival and failure-free survival by treatment and metastatic burden HR=hazard ratio. Solid lines show the Kaplan-Meier analysis and dotted lines show the flexible parametric model.

Figure 3 shows prespecified and exploratory subgroup analyses. In the analysis by metastatic burden, overall survival was improved in patients with low metastatic burden at baseline who were allocated radiotherapy (HR 0·68, 95% CI 0·52–0·90; p=0·007; 3-year survival 73% with control *vs* 81% with radiotherapy; table 2), with no evidence of non-proportional hazards. We found some evidence of heterogeneity of treatment effect by metastatic burden (interaction p=0·0098; figure 4). In patients with a high metastatic burden, there was no evidence of a treatment effect (HR 1·07, 95% CI 0·90–1·28; p=0·420). The appendix (p 7) shows further exploratory consistency-of-effect analyses.

758 failure-free survival events were reported in patients assigned to the control group, largely driven by rising PSA (appendix p 3); median failure-free survival was 13 months (IQR 6–33) and 3-year failure-free survival was 23%. In patients assigned to the radiotherapy group, 685 failure-free survival events were reported, with median failure-free survival of 17 months (IQR 8–53) and 3-year failure-free survival of 32%. Overall, failure-free survival was improved with radiotherapy (HR 0·76, 95% CI 0·68–0·84; p<0·0001; figure 2B). There was some evidence of non-proportional hazards (p=0·066). An analysis of restricted mean survival time found mean failure-free survival, restricted to the first 59 months on trial, was 21·4 months in the control group, compared with 26·2 months with radiotherapy (difference 4·8 months, 95% CI 2·8–6·7; p<0·0001; table 2).

In the prespecified subgroup analysis by metastatic burden, failure-free survival was improved in patients with low metastatic burden at baseline who were allocated radiotherapy (HR 0·59, 95% CI 0·49–0·72; p<0·0001; table 2). Evidence of a differential treatment effect from radiotherapy compared with the high metastatic burden subgroup was also noted (interaction p=0·002; HR 0·88, 95% CI 0·77–1·01; p=0·059; figure 4). The appendix (p 8) shows prespecified and exploratory consistency-of-effect analyses.

643 (84%) of 761 deaths were attributed to prostate cancer (329 [84%] of 391 in the control group and 314 [85%] of 370 in the radiotherapy group). Adjusted competing risks regression for prostate cancer-specific survival using the Fine and Gray method provided no evidence of an overall treatment effect (sub-HR 0·94, 95% CI 0·81–1·10; robust p=0·431; table 2). There was evidence of an effect in patients with low metastatic burden (sub-HR 0·65, 95% CI 0·47–0·90; robust p=0·010), but no evidence of a treatment effect was noted in patients with high metastatic burden (1·11, 0·92–1·33; robust p=0·279). A significant interaction was seen between treatment effect and metastatic burden (robustly estimated interaction p=0·007).

The appendix (pp 9, 10) shows the analysis of progression-free survival for all patients and by baseline metastatic burden. A treatment effect was only noted in patients with a low metastatic burden (HR 0·78, 95% CI 0·63–0·98; p=0·033; table 2).

One or more symptomatic local events were reported by 432 (42%) of 1029 patients allocated to the control group compared with 450 (44%) of 1032 patients assigned to the radiotherapy group. There was no evidence of a difference in time to first symptomatic local event by treatment allocation (HR 1·07, 95% CI 0·93–1·22; p=0·349; table 2). Table 3 shows the number of patients reporting each type of symptomatic local event at least once.

**Table 3:** Incidence of symptomatic local events reported before and after treatment period

	**Within treatment window**		**After treatment window**	
	Control (n=1029)	Radiotherapy (n=1032)	Control (n=1029)	Radiotherapy (n=1032)
Transurethral resection of the prostate	9 (1%)	13 (1%)	23 (2%)	24 (2%)
Ureteric stent	5 (<1%)	3 (<1%)	16 (2%)	7 (1%)
Surgery for bowel obstruction	0 (0%)	0 (0%)	0 (0%)	1 (<1%)
Urinary catheter	14 (1%)	18 (2%)	35 (3%)	36 (3%)
Nephrostomy	2 (<1%)	2 (<1%)	8 (1%)	3 (<1%)
Colostomy	0 (0%)	0 (0%)	2 (<1%)	1 (<1%)
Acute kidney injury	2 (<1%)	6 (1%)	31 (3%)	35 (3%)
Urinary tract infection	14 (1%)	31 (3%)	49 (5%)	75 (7%)
Urinary tract obstruction	4 (<1%)	7 (1%)	24 (2%)	17 (2%)
Prostate cancer death	2 (<1%)	1 (<1%)	327 (32%)	313 (30%)

Treatment window defined as 12 weeks from randomisation for patients in either treatment group who did not receive docetaxel, and 28 weeks from randomisation for those who did.

There was some evidence of heterogeneity in the effect on failure-free survival by nominated radiotherapy schedule (interaction p=0·072; appendix p 8). Prespecified analyses in 1082 patients who nominated the daily schedule before randomisation (55 Gy in 20 fractions over 4 weeks) found strong evidence of a failure-free survival advantage with radiotherapy compared with control (HR 0·69, 95% CI 0·59–0·80; p<0·0001). Among these 1082 patients, 212 deaths were reported in the control group and 188 in the radiotherapy group (stratified log-rank p=0·123; HR 0·86, 95% CI 0·71–1·05; p=0·128). There was insufficient evidence of a difference in failure-free survival in 979 patients who nominated the weekly radiotherapy schedule (36 Gy in six fractions over 6 weeks; HR 0·85, 95% CI 0·73–0·99; p=0·033) to report on survival.

Adverse effects on the RTOG scale during radiotherapy were modest, with 48 (5%) of 920 patients allocated radiotherapy who started radiotherapy and who completed at least one acute toxicity form reporting grade 3 or 4 adverse events; 43 (5%) patients reported their worst acute bladder toxic effect as grade 3 or 4, and eight (1%) reported their worst acute bowel toxic effect as grade 3 or 4 (table 4; appendix p 11); no grade 5 toxic effects were reported. The incidence of acute bladder and bowel effects (grade 1–4) was lower for those who nominated the weekly radiotherapy schedule than for those who nominated the daily schedule (282 [65%] bladder and 206 [47%] bowel *vs* 341 [71%] bladder and 297 [62%] bowel). Patients in both control and radiotherapy groups reported a low incidence of grade 3 and 4 RTOG late effects (one [1%] control *vs* 37 [4%] radiotherapy; table 5).

**Table 4:** Worst reported acute radiotherapy bladder and bowel toxic effect (RTOG scale) in patients allocated radiotherapy

	**Weekly schedule (n=437)**	**Daily schedule (n=483)**	**Total (n=920)**
**Bladder**			
Grade 0	152 (35%)	142 (29%)	294 (32%)
Grade 1 or 2	262 (60%)	318 (66%)	580 (63%)
Grade 3 or 4	20 (5%)	23 (5%)	43 (5%)
Missing	3	0	3
**Bowel**			
Grade 0	231 (53%)	185 (38%)	416 (45%)
Grade 1 or 2	205 (47%)	290 (60%)	495 (54%)
Grade 3 or 4	1 (<1%)	7 (1%)	8 (1%)
Missing	0	1	1

RTOG=Radiation Therapy Oncology Group.

**Table 5:** Grade 3 or 4 worst late radiotherapy toxicity score (RTOG scale) in patients who received radiotherapy (for research or progression)

	Control (n=187)*	**Radiotherapy (n=988)**
Diarrhoea	1 (1%)	12 (1%)
Proctitis	0 (0%)	11 (1%)
Cystitis	0 (0%)	7 (1%)
Haematuria	0 (0%)	6 (1%)
Rectal–anal stricture	0 (0%)	0 (0%)
Urethral stricture	0 (0%)	4 (<1%)
Rectal ulcer	0 (0%)	0 (0%)
Bowel obstruction	0 (0%)	1 (<1%)

Treatment groups correspond to the safety population. There were no reported grade 5 late radiotherapy toxic events. RTOG=Radiation Therapy Oncology Group.

* Relates to patients assigned control who had some radiotherapy at some point.

The proportion of patients in the safety population reporting at least one severe adverse event of CTCAE grade 3 or worse was similar in both study groups and was dominated by side-effects associated with long-term hormone therapy (398 [38%] of 1050 in the control group and 380 [39%] of 985 in the radiotherapy group; appendix pp 4, 12); with no evidence of a difference in time to first grade 3 or worse event (HR 1·01, 95% CI 0·87–1·16; p=0·941). In 2028 patients with adverse event data at approximately 6 months, the proportions reporting a grade 3 or worse adverse event were similar (225 [21%] of 1047 in the control group and 212 [22%] of 981 in the radiotherapy group). Of 1125 patients with adverse event data at 1 year, 63 (12%) of 531 patients in the control group and 78 (13%) of 594 in the radiotherapy group reported a grade 3 or worse adverse event. At 2 years, of 533 patients with data available, 37 (15%) of 240 in the control group and 37 (13%) of 293 in the radiotherapy group reported a grade 3 or worse event. The pattern and levels of adverse events were very similar when considering the intention-to-treat population (data not shown). No deaths were reported as related to the research treatment.

**Figure 5:**
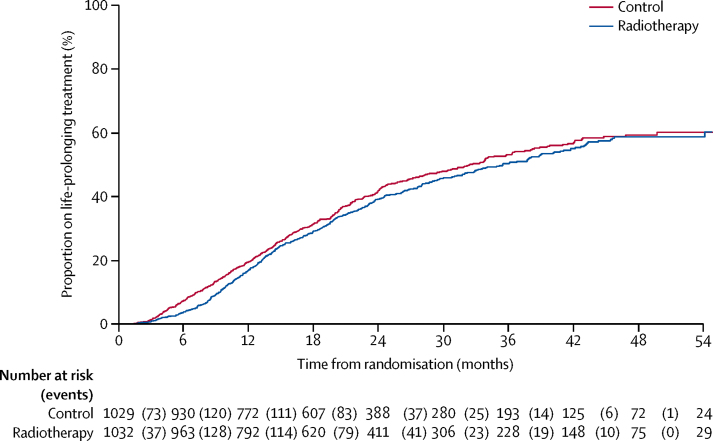
Time from randomisation to life-prolonging therapy

The appendix (pp 13, 14) shows time to first new treatment after failure-free survival event and time to first life-prolonging therapy (defined as available agents with proven survival gain in castration-resistant prostate cancer: docetaxel, abiraterone, cabazitaxel, enzalutamide, and radium 223). There was no evidence of any difference in time to any therapy, but an indication that patients allocated radiotherapy received a life-prolonging treatment sooner after disease progression but later after randomisation than did patients allocated control (figure 5; appendix pp 13, 14). Overall exposure to treatment for progression is summarised in the appendix (p 5).

## Discussion

This randomised comparison of more than 2000 patients with metastatic prostate cancer showed that local radiotherapy to the prostate did not improve overall survival for unselected patients. However, a prespecified analysis showed that prostate radiotherapy did improve overall survival (from 73% to 81% at 3 years) in those with a low metastatic burden, which represented 40% of the comparison population.

Our subgroup finding meets all criteria proposed by Sun and colleagues to assess credibility of subgroup effects:^20^ low metastatic burden status was determined from scans taken before randomisation; the hypothesis—including the direction of the effect—was specified a priori; only a few hypothesised subgroup effects were tested; the interaction test suggested a low likelihood that the apparent subgroup effect could be accounted for by chance; the subgroup effect was independent of other assessed variables; the size of the subgroup effect was large (HR 0·68 for low metastatic burden and HR 1·07 high metastatic burden); and the interaction was consistent both with other related outcome measures in STAMPEDE (eg, failure-free survival) and with the interaction reported on number of bone metastases in the HORRAD trial^12^ (less than five bone metastases, HR 0·68; five or more bone metastases, HR 1·06). It also seems plausible that the effect of local radiotherapy would be diminished in patients with a greater burden of metastatic disease. There is, therefore, good reason to accept that prostate radiotherapy improves survival of men with a low metastatic burden and that it should now be a standard treatment. Unlike many other new interventions for metastatic cancer, prostate radiotherapy does not require regulatory approval and is readily available at modest cost in most parts of the world.

When this comparison was designed, the standard radical radiotherapy dose schedule for localised prostate cancer was 74 Gy in 37 fractions over 7·5 weeks. It was felt that this schedule would be too burdensome for patients with metastatic disease, and the two more convenient schedules permitted in the trial were chosen based on a survey of investigators' opinions. The trial has proven the principle that local radiotherapy can improve survival, but the optimum dose schedule and technique are uncertain. Radical radiotherapy for localised prostate cancer is now typically given to a dose of 60 Gy in 20 fractions over 4 weeks.^21^ With contemporary techniques for target delineation and treatment delivery, this schedule is well tolerated^21^ and might be expected to be at least as effective as the two schedules tested in the trial.

It is well known that prostate radiotherapy improves survival for men with locally advanced (T3–4 N0 M0) prostate cancer.^22^ We have now found that prostate radiotherapy also improves survival for men with a low metastatic burden (T_any_ N_any_ M1) prostate cancer. It therefore seems safe to conclude that radiotherapy would also improve survival for men with pelvic node-positive prostate cancer (T_any_ N1 M0). This is important, because it is not feasible to do randomised trials specifically in men with non-metastatic node-positive prostate cancer and because such men often receive systemic treatment alone. In the current study, roughly 60% of patients were N1 in both the high and low metastatic burden subgroups. The benefit we have shown for prostate radiotherapy in prostate cancer with a low metastatic burden also raises another question: would there be further benefit from additional radiotherapy to the oligometastases themselves?

Low metastatic burden disease is sometimes known as oligometastatic. Although this term is widely used, it is imprecise and potentially misleading because it implies only a small number of metastases. Patients with low metastatic burden disease, according to the CHAARTED definition, may have an unlimited number of metastases provided they are confined to lymph nodes and the axial skeleton.

Our data have several strengths to note. This is a large randomised dataset with broad engagement from more than 100 hospitals across Switzerland and the UK. By incorporating the comparison into the established STAMPEDE protocol, following peer-review and protocol amendment, we recruited to an enlarged target well ahead of schedule (2061 patients in 3·5 years rather than 1250 patients in 4 years).

Our data also have some limitations. First, the possible clinical relevance of metastatic burden in patients with prostate cancer only became widely apparent when the CHAARTED trial reported.^15^ We determined metastatic burden by retrospectively collecting retrievable baseline scans. This was possible in most (94%) but not all patients. Second, compliance with allocation to prostate radiotherapy was not complete (94%) and this could underestimate the true effect size for radiotherapy. Third, median follow-up (37 months) is shorter than median survival (46 months); this could be particularly relevant to the analysis of symptomatic local events, which can occur late and after disease progression. We plan to continue follow-up and to link to routinely collected electronic health records to capture symptomatic local events. Our current analysis indicates that radiotherapy does not improve survival for patients with a high metastatic burden. Future analyses will explore whether prostate radiotherapy might still be useful in such patients for prevention of symptomatic local events. Fourth, up-front systemic treatment of metastatic prostate cancer has evolved. Most patients in this comparison received androgen deprivation alone. Docetaxel was permitted in addition to hormone therapy after its approval in the UK^15^, ^23^, ^24^ and was used, therefore, mostly in recently randomised patients who had the shortest follow-up. Although only roughly one in six patients received docetaxel in this comparison, there is no evidence to suggest that prostate radiotherapy is more or less effective when docetaxel is given in addition to androgen deprivation therapy. More recently, abiraterone has become another option in this setting.^25^, ^26^, ^27^, ^28^ The value of prostate radiotherapy in men receiving abiraterone is being tested in the PEACE1 trial (NCT01957436),^29^ and the prospectively planned STOPCAP M1 meta-analysis of these trials will explore this further.^30^

We have tested local treatment to the prostate using radiotherapy. It is possible that other forms of local treatment—such as radical prostatectomy—might also be effective. If the benefit of radiotherapy is mediated by local tumour eradication, one would expect surgery to be at least as effective. However, radiotherapy might be effective via other mechanisms (eg, immune modulation), so the role of surgery in men with metastatic prostate cancer remains unproven. The feasibility of prostate surgery in this setting is being tested in the g-RAMMP trial (NCT02454543) and the TROMBONE feasibility study.^31^

There is uncertainty regarding the optimum definition of low metastatic burden (oligometastatic) prostate cancer. We used the same definition as that in the CHAARTED trial, but this is not necessarily the optimum definition. Our findings were almost identical when applying a variant of the definition used in the LATITUDE trial;^25^ absence of visceral metastases and fewer than three bone metastases (data not shown). Exploratory analyses of a broader cohort of patients in the STAMPEDE study will inform the definition of oligometastatic disease, with the aim of refining patients' selection for prostate radiotherapy. All current definitions are based on conventional imaging using CT and bone scans. Caution will be required in extrapolating these results to patients imaged with more sensitive techniques (eg, PSMA PET). For example, patients with low metastatic burden on conventional imaging should not be denied prostate radiotherapy because they have additional lesions identified on a PET scan.

In summary, radiotherapy to the prostate did not improve survival for unselected patients with newly diagnosed metastatic prostate cancer, but, in a prespecified subgroup analysis, overall survival did improve in men with a low metastatic burden. Therefore, prostate radiotherapy should be a standard treatment option for men with a low metastatic burden. These findings also raise the possibility that local treatment to the primary tumour should be explored for patients with small-volume metastatic disease from other malignant diseases.
