# Supplementary material for: Radiotherapy to the primary tumour for newly diagnosed, metastatic prostate cancer (STAMPEDE): a randomised controlled phase 3 trial
Source: Lancet. 2018 Dec 1;392(10162):2353–66. doi: 10.1016/S0140-6736(18)32486-3 (PMC6269599; doi:10.1016/S0140-6736(18)32486-3)
Supplement: Supplementary appendix [file mmc1.pdf]

THE LANCET

Supplementary appendix

This appendix formed part of the original submission and has been peer reviewed. We post it as supplied by the authors.

Supplement to: Parker CC, James ND, Brawley CD, et al. Radiotherapy to the primary tumour for newly diagnosed, metastatic prostate cancer (STAMPEDE): a randomised controlled phase 3 trial. *Lancet* 2018; published online Oct 21. http://dx.doi.org/10.1016/S0140-6736(18)32486-3.

Supplementary Material

Supplementary Table 1: Baseline Characteristics For Metastatic Volume Analyses

Characteristic		SOC		SOC+RT	
		**Lower metastatic burden (n=409)**	**Higher metastatic burden (n=567)**	**Lower metastatic burden (n=410)**	**Higher metastatic burden (n=553)**
Age at randomisation (years)	Median (IQR)	68 (63-73)	68 (63-73)	68 (63-73)	68 (63-73)
	Range	44-83	37-86	45-84	46-87
WHO Performance Status	0	305 (75%)	390 (69%)	313 (76%)	376 (68%)
	1-2	104 (25%)	177 (31%)	97 (24%)	177 (32%)
Pain from prostate cancer	Absent	361 (90%)	423 (75%)	363 (90%)	437 (80%)
	Present	42 (10%)	141 (25%)	39 (10%)	108 (20%)
	Missing	6	3	8	8
Previous notable health issues*	Myocardial infarction	25 (6%)	35 (6%)	19 (5%)	37 (7%)
	Cerebrovascular disease	14 (3%)	13 (2%)	8 (2%)	18 (3%)
	Congestive heart failure	5 (1%)	0 (0%)	3 (1%)	5 (1%)
	Angina	23 (6%)	21 (4%)	18 (4%)	31 (6%)
	Hypertension	168 (41%)	222 (39%)	179 (44%)	238 (43%)
T-category at randomisation	T0	0 (0%)	0 (0%)	1 (<1%)	0 (0%)
	T1	7 (2%)	4 (1%)	6 (2%)	5 (1%)
	T2	39 (10%)	39 (8%)	32 (8%)	50 (10%)
	T3	250 (64%)	305 (61%)	261 (66%)	302 (61%)
	T4	95 (24%)	151 (30%)	94 (24%)	138 (28%)
	TX	18	68	16	58
N-category at randomisation	N0	144 (37%)	188 (36%)	142 (36%)	187 (38%)
	N+	249 (63%)	333 (64%)	258 (65%)	311 (62%)
	NX	16	46	10	55
Sites of metastases	Bone	311 (76%)	561 (99%)	311 (76%)	549 (99%)
	Liver	0 (0%)	22 (4%)	0 (0%)	18 (3%)
	Lung	0 (0%)	36 (6%)	0 (0%)	41 (7%)
	Distant lymph nodes	140 (34%)	137 (24%)	149 (36%)	128 (23%)
	Other	18 (4%)	16 (3%)	17 (4%)	15 (3%)
Gleason sum score	<=7	77 (19%)	84 (15%)	84 (21%)	81 (15%)
	8-10	321 (81%)	460 (85%)	308 (79%)	449 (85%)
	Unknown	11	23	18	23
PSA before androgen deprivation therapy (ng/ml)	Median (IQR)	48 (19-120)	181 (60-619)	55 (23-138)	180 (52-668)
	Range	2-5560	1-20590	1-1706	2-11156
Time from diagnosis (Days)	Median (IQR)	81 (63-103)	69 (49-86)	80 (59-101)	69 (52-88)
	Range	6-2297	0-3495	9-1276	0-821
	Missing	5	1	5	9
Days from starting hormones	Median (IQR)	49 (31-67)	54 (37-71)	52 (31-68)	58 (37-73)
	Range	-32;84	-32;84	-10;85	-7;84
	Missing	0	0	0	1
Planned SOC docetaxel	No	342 (84%)	462 (81%)	348 (85%)	444 (80%)
	Yes	67 (16%)	105 (19%)	62 (15%)	109 (20%)
Nominated RT schedule	36Gy/6f/6wk	190 (46%)	257 (45%)	168 (41%)	291 (53%)
	55Gy/20f/4wk	219 (54%)	310 (55%)	242 (59%)	262 (47%)

* Data missing for 4 SOC and 6 SOC+RT patients (lower metastatic burden), and 2 SOC and 1 SOC+RT patients (higher metastatic burden).

Note: This table excludes the 122 patients in whom metastatic burden could not be determined."

Supplementary Table S2: Radiotherapy Treatment Summary

Characteristic		SOC (n=1029)	SOC+RT (n=1032)
		**N (%)**	**N (%)**
Started RT within 1 year of randomisation	Yes	20 (2%)	968 (94%)
	No	1009 (98%)	64 (6%)
RT schedule received, if started	Nominated RT schedule	5 (25%)	906 (94%)
	Alternative protocol RT schedule	1 (5%)	36 (4%)
	Non-protocol RT schedule	14 (70%)	26 (3%)
		**Median (IQR)**	**Median (IQR)**
RT timing	Randomisation to starting RT (days)	193 (128-249)	35 (28-60)
	Starting androgen deprivation therapy to starting RT (days)	257 (176-310)	95 (74-120)

Note: RT schedule was nominated before randomisation so patients allocated SOC also have a nominated RT schedule even though not allocated to receive it

Supplementary Table S3: Worst Component of First Failure-Free Survival Event Reported; All Patients Who Reported Progression

**Contributing Event**	**SOC (n=758)**	**SOC+RT (n=685)**
PCa-related death	12 (2%)	17 (2%)
Distant metastases	63 (8%)	85 (12%)
Lymph node involvement	6 (1%)	8 (1%)
Skeletal-related event	16 (2%)	11 (2%)
Local progression	19 (3%)	12 (2%)
PSA failure	642 (85%)	552 (81%)

Supplementary Table S4: Grade 3-5 AEs for Selected Body System Categories (CTCAE)

**Toxicity Category**	**SOC (n=1050)**	**SOC+RT (n=985)**
Endocrine disorder	152 (14%)	140 (14%)
Musculoskeletal disorder	90 (9%)	91 (9%)
Renal disorder*	43 (4%)	49 (5%)
Blood / bone marrow	50 (5%)	37 (4%)
Lab abnormalities	39 (4%)	39 (4%)
Gastrointestinal disorder**	35 (3%)	35 (4%)

Note: Treatment arms correspond to safety population.
* 1 grade 5 renal toxicity was reported: SOC: 0 (0%), SOC+RT: 1 (<1%)
** 2 grade 5 gastrointestinal toxicities were reported: SOC: 1 (<1%), SOC+RT: 1 (<1%)

Note: Ordered by descending total frequency

CTCAE = common terminology criteria for adverse events

Supplementary Table S5: Summary of Treatments Reported for Progression

Treatment Type	SOC (n=758)	SOC+RT (n=685)	Total (n=1443)
**Life-Prolonging Treatment**			
Abiraterone	158 (21%)	139 (20%)	297 (21%)
Cabazitaxel	45 (6%)	43 (6%)	88 (6%)
Docetaxel	249 (33%)	226 (33%)	475 (33%)
Enzalutamide	243 (32%)	247 (36%)	490 (34%)
Radium-223	65 (9%)	53 (8%)	118 (8%)
**Other Treatment**			
Anti-androgens	476 (63%)	395 (58%)	871 (60%)
Cox-2 inhibition	0 (0%)	1 (<1%)	1 (<1%)
Dexamethasone	138 (18%)	158 (23%)	296 (21%)
Other bisphosphonate	8 (1%)	6 (1%)	14 (1%)
Other chemotherapy	8 (1%)	4 (1%)	12 (1%)
Prednisolone / prednisone	112 (15%)	107 (16%)	219 (15%)
Stilboestrol	18 (2%)	21 (3%)	39 (3%)
Strontium	1 (<1%)	0 (0%)	1 (<1%)
Zoledronic acid	104 (14%)	78 (11%)	182 (13%)

Note: Ordered alphabetically within grouping

Supplementary Figure S1: Patients in "M1|RT comparison" in context of all accrual to STAMPEDE

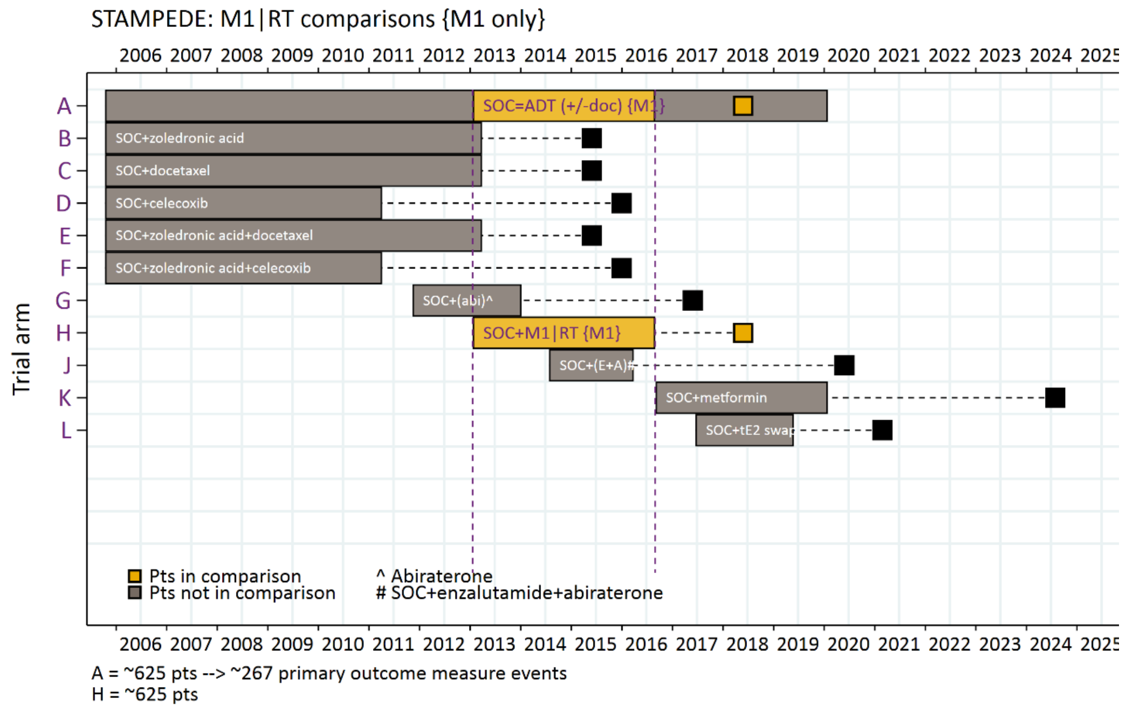

Key

- Yellow bars: patients contributing to this comparison, newly-diagnosed metastatic patients contemporaneously randomised to arm A (SOC) or arm H (SOC+RT)
- Grey bars: patients in the STAMPEDE protocol not contributing to this comparison
- Small yellow squares: timing of primary survival analysis for this comparison
- Small black squares: timing of primary survival analyses for other comparisons

Supplementary Figure S2: Forest Plot of Exploratory Treatment Effect on Overall Survival Within Other Selected Baseline Categories

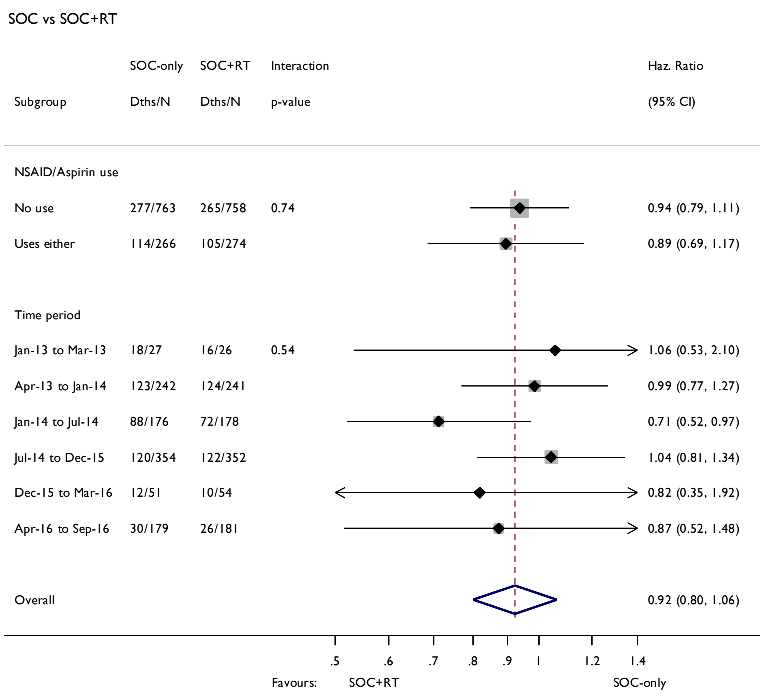

Note: Time periods are defined by the other protocol arms which are open to recruitment contemporaneously; see Supplementary Figure S1

Supplementary Figure S3: Forest Plot of Treatment Effect on Failure-Free Survival Within Selected Baseline Categories

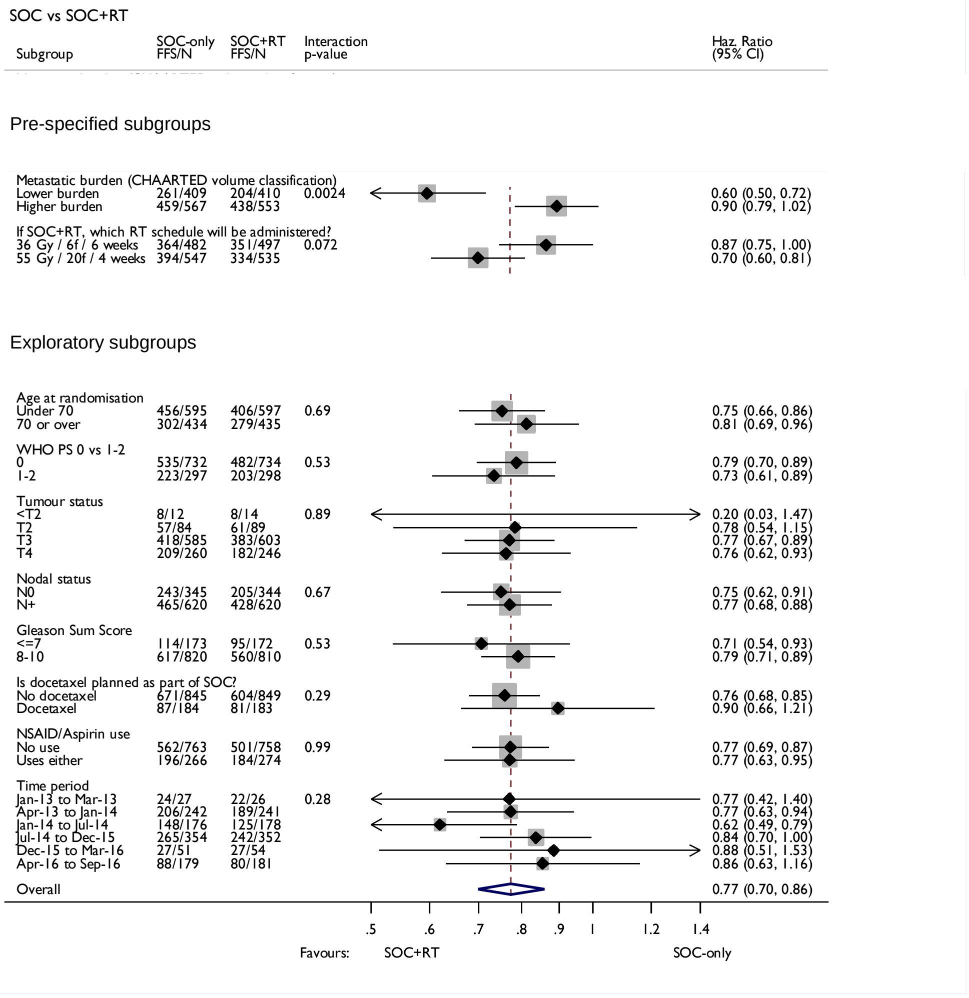

Supplementary Figure S4: Progression-free survival

-- S4A: Progression-Free Survival – all patients

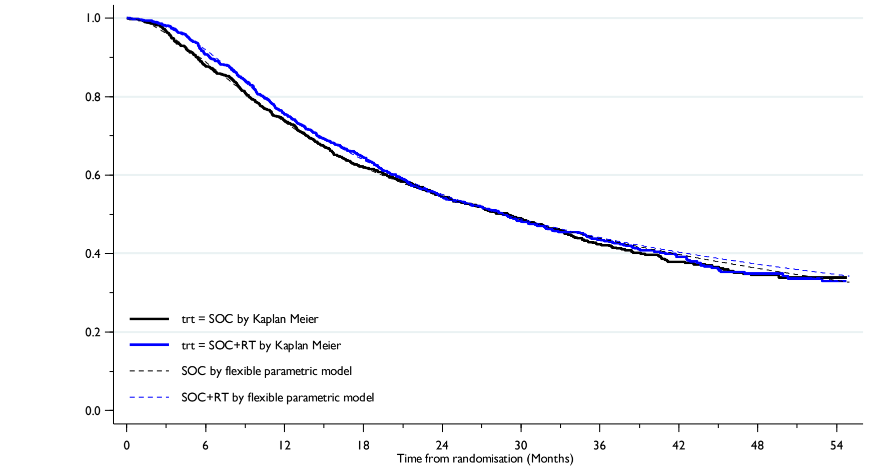

Number of patients (events)

SOC	1029	(124)	886	(140)	737	(117)	598	(65)	408	(38)	301	(37)	208	(20)	138	(10)	73	(1)	25
SOC+RT	1032	(94)	914	(153)	749	(106)	606	(87)	410	(44)	308	(27)	229	(20)	143	(13)	74	(3)	31

HR 0.96 (95%CI 0.85-1.08; p=0.468)

-- S4B: Progression-Free Survival – lower metastatic burden patients

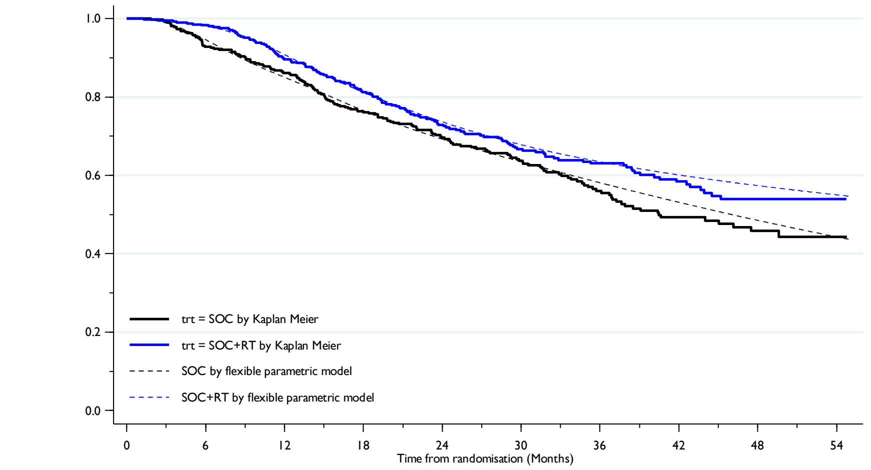

Number of patients (events)

SOC	409	(29)	373	(27)	340	(38)	291	(23)	204	(16)	156	(17)	108	(12)	73	(4)	39	(1)	14
SOC+RT	410	(7)	399	(35)	361	(33)	306	(29)	226	(18)	179	(9)	142	(9)	94	(6)	50	(0)	20

HR 0.78 (95%CI 0.63-0.98; p=0.033)

-- S4C: Progression-Free Survival – higher metastatic burden patients

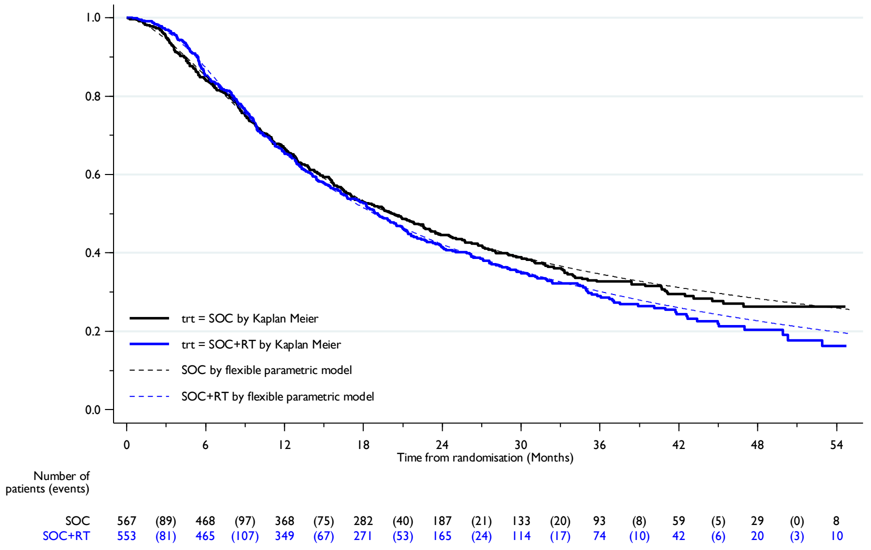

HR 1.09 (95%CI 0.94-1.26; p=0.252)

Supplementary Figure S5: Acute Toxicity During Radiotherapy

- S5A: Bladder toxicity

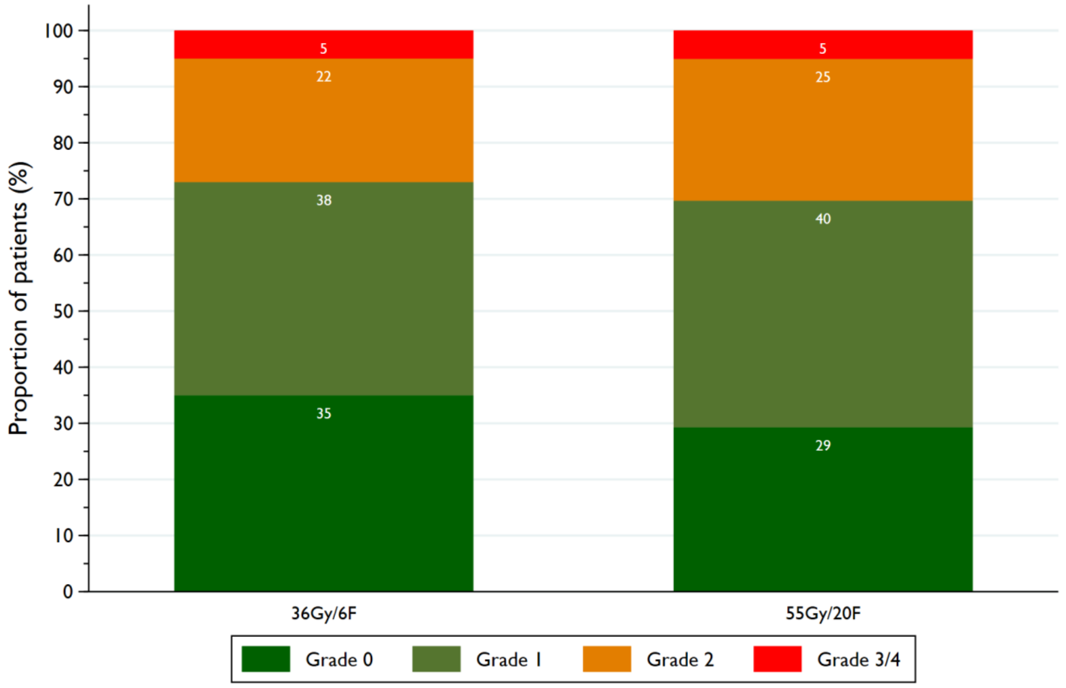

Note: Proportions based on 434 patients who received or were planned for 36Gy/6f and 483 patients for 55Gy/20f.

-- S5B: Bowel toxicity

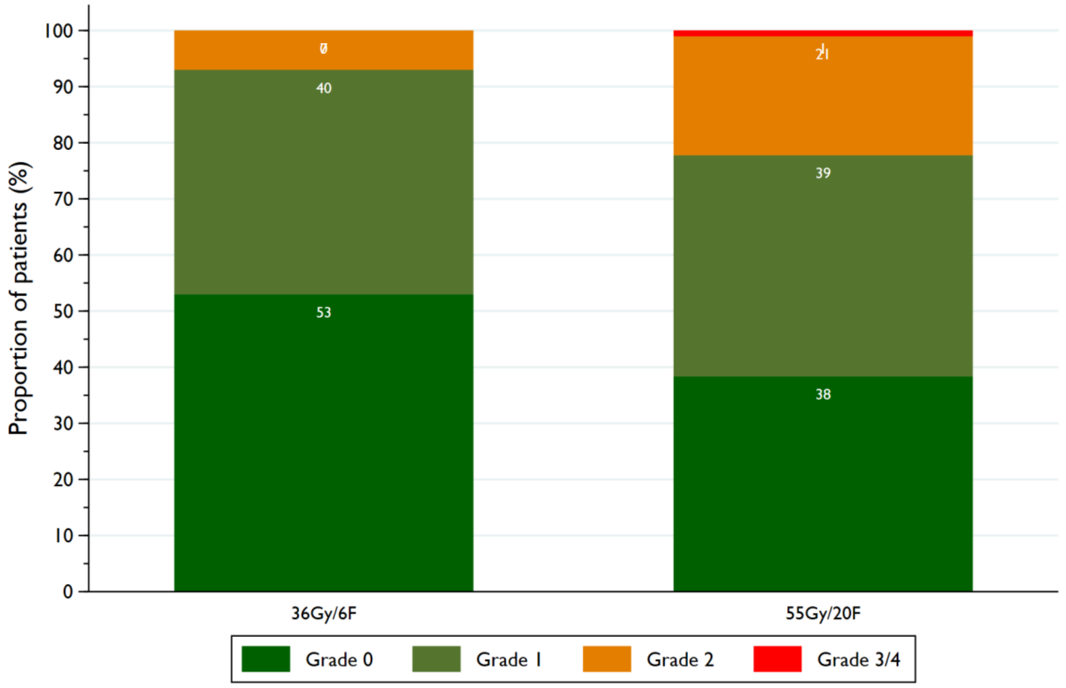

Note: Proportions based on 437 patients who received or were planned for 36Gy/6f and 482 patients for 55Gy/20f.

Supplementary Figure S6: Worst Ever Reported CTCAE V.4 Toxicity

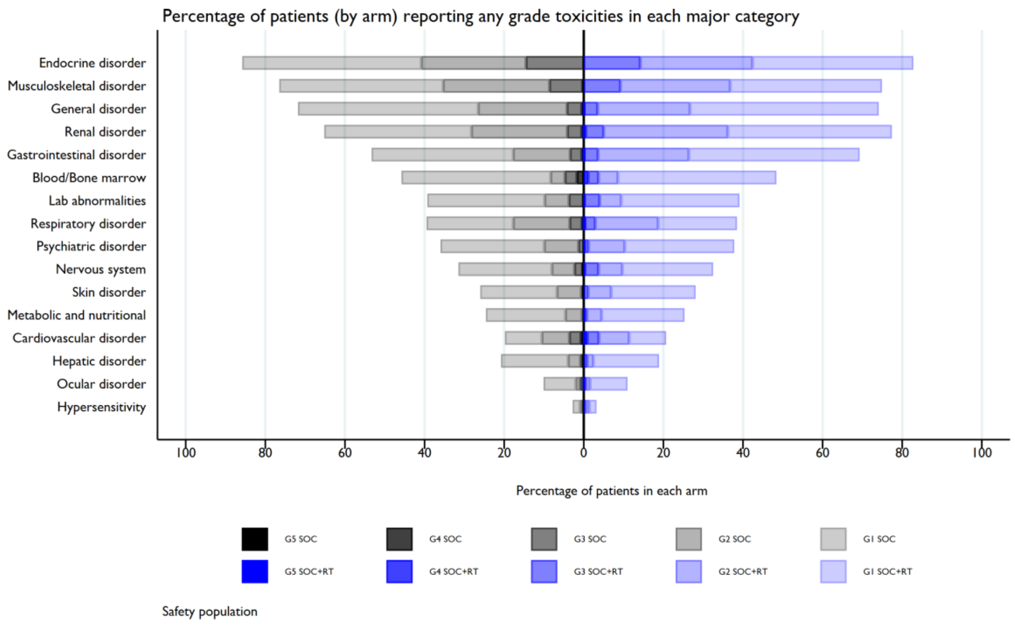

Supplementary Figure S7: Time from failure-free survival event to subsequent treatment

-- S7A: Time from failure-free survival event to any treatment

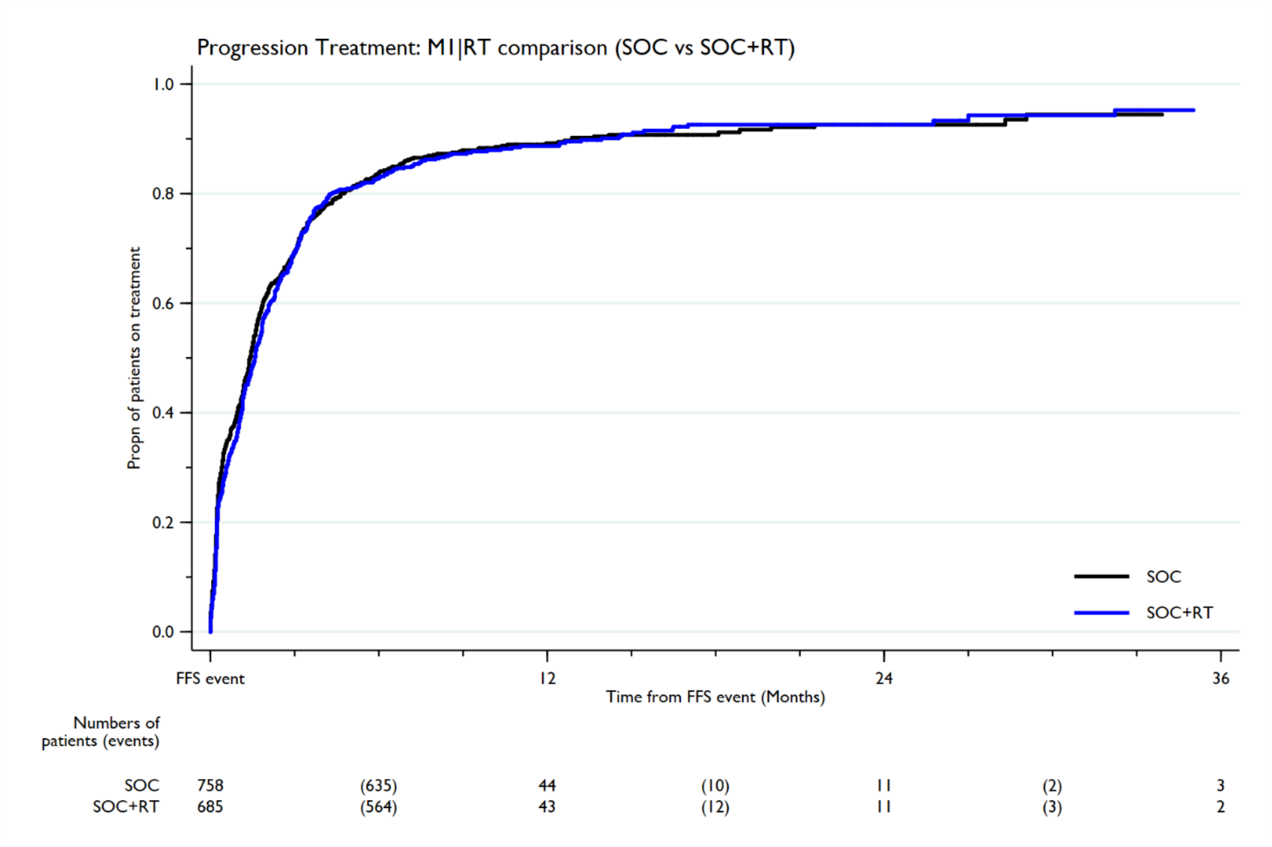

-- S7B: Time from failure-free survival event to “life-prolonging” therapy

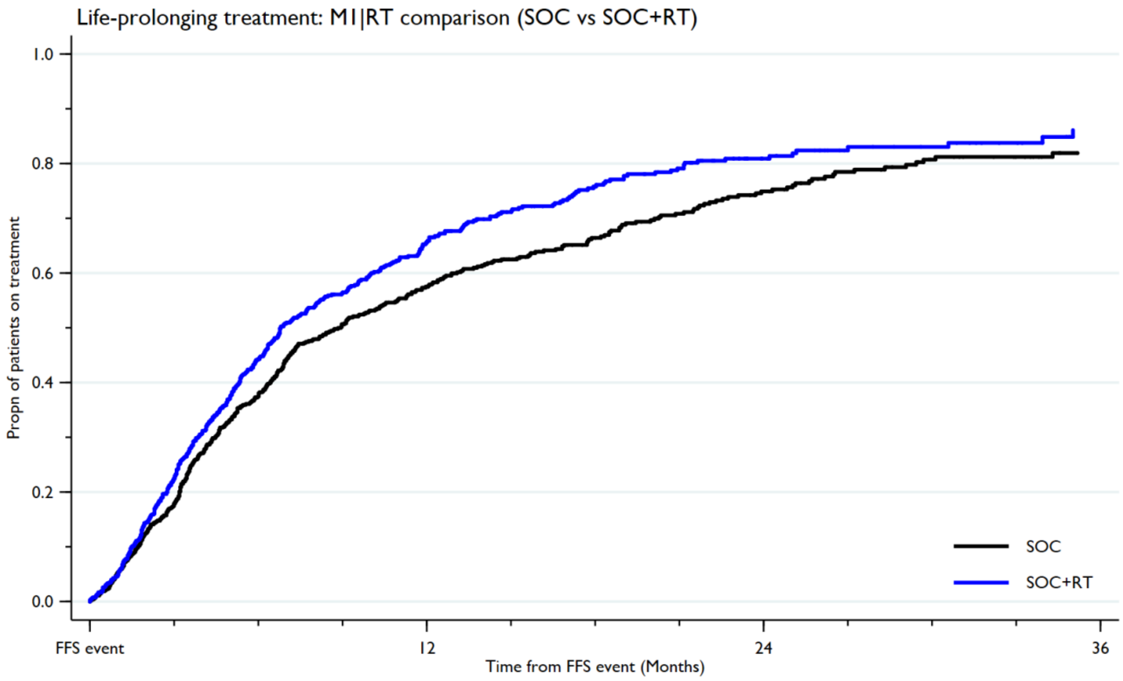

Numbers of patients (events)

SOC	758	(383)	212	(69)	70	(16)	23
SOC+RT	685	(389)	160	(54)	42	(7)	10
